# ^18^F-FDG PET/CT radiomic analysis and artificial intelligence to predict pathological complete response after neoadjuvant chemotherapy in breast cancer patients

**DOI:** 10.1007/s11547-025-01958-4

**Published:** 2025-01-28

**Authors:** Luca Urso, Luigi Manco, Corrado Cittanti, Sara Adamantiadis, Klarisa Elena Szilagyi, Giovanni Scribano, Noemi Mindicini, Aldo Carnevale, Mirco Bartolomei, Melchiore Giganti

**Affiliations:** 1 Department of Translational Medicine, University of Ferrara, Ferrara, Italy; 2 Nuclear Medicine Unit, Onco-Hematology Department, University Hospital of Ferrara, Via Aldo Moro 8, 44124 Ferarra, Italy; 3 Medical Physics Unit, University Hospital of Ferrara, Ferrara, Italy; 4 Department of Physics and Earth Science, University of Ferrara, Ferrara, Italy; 5 Oncology Unit, University Hospital of Ferrara, Ferrara, Italy

**Keywords:** Breast cancer, Neoadjuvant chemotherapy, ^18^F-FDG, PET/CT, Radiomics, Machine learning, Artificial intelligence

## Abstract

**Purpose:**

Build machine learning (ML) models able to predict pathological complete response (pCR) after neoadjuvant chemotherapy (NAC) in breast cancer (BC) patients based on conventional and radiomic signatures extracted from baseline [^18^F]FDG PET/CT.

**Material and methods:**

Primary tumor and the most significant lymph node metastasis were manually segmented in baseline [^18^F]FDG PET/CT of 52 newly diagnosed BC patients. Clinical parameters, NAC and conventional semiquantitative PET parameters were collected. The standard of reference considered was surgical pCR after NAC (ypT0;ypN0). Eight-hundred-fifty-four radiomic features (RFts) were extracted from both PET and CT datasets, according to IBSI; robust RFTs were selected. The cohort was split in training (70%) and validation (30%) sets. Four ML Models (Clinical Model, CT Model, PET Model_T and PET Model_T + N) each one with 3 learners (Random Forest (RF), Neural Network and Stochastic Gradient Descendent) were trained and tested using RFts and clinical signatures. PET Models were built considering robust RFTs extracted from either primary tumor alone (PET Model_T) or also including the reference lymph node (PET Model_T + N).

**Results:**

72 pathological uptakes (52 primary BC and 20 lymph node metastasis) at [^18^F]FDG PET/CT were segmented. pCR occurred in 44.2% cases. Twelve, 46 and 141 robust RFts were selected from CT Model, PET Model_T and PET Model_T + N, respectively. PET Models showed better performance than CT and Clinical Models. The best performances were obtained by the RF algorithm of the PET Model_T + N (AUC = 0.83;CA = 0.74;TP = 78%;TN = 72%).

**Conclusion:**

ML models trained on PET/CT radiomic features extracted from primary BC and lymph node metastasis could concur in the prediction of pCR after NAC and improve BC management.

**Supplementary Information:**

The online version contains supplementary material available at 10.1007/s11547-025-01958-4.

## Introduction

Breast Cancer (BC) is still the leading neoplasm in terms of incidence and of cancer-related death in women worldwide [1]. The introduction of neoadjuvant chemotherapy (NAC) has improved the outcomes of BC patients [2, 3]. Literature evidence largely demonstrated that pathological complete response (pCR) after NAC is an early indicator of long-term outcomes [4]. Patients achieving pCR after NAC have significantly longer event-free survival (EFS) and overall survival (OS), in particular in aggressive BC subtypes, such as triple-negative breast cancer (TNBC) [5]. Therefore, the prediction of pCR after NAC through imaging represents a clinically relevant need, as it may improve management and enable treatment personalization. However, available imaging modalities do not guarantee optimal results to solve this task yet. Breast magnetic resonance imaging (MRI) has high accuracy in defining the response to NAC of primary BC [6]. However, breast MRI does not allow whole body staging and de-novo oligometastatic disease could remain concealed. Moreover, false positive (FP) and false negative (FN) findings have been described and the performances are poorer in Luminal subtypes [7–10]. 2-deoxy-2-[^18^F]fluoro-D-glucose ([^18^F]FDG) Positron Emission Tomography / Computed Tomography (PET/CT) has been investigated in the last 10 years for imaging BC patients and recently included into guidelines also for the assessment of response to NAC in TNBC and human epidermal growth factor receptor 2 (HER-2) enriched BC [11–13]. However, also [^18^F]FDG PET/CT has limits and the accuracy of conventional PET parameters in the prediction of pCR could be unsatisfactory [14].

Several studies in the last few years focused on the potential information derivable from baseline imaging through the use of radiomic analysis combined with artificial intelligence (AI) in various oncological diseases, including BC [15–18]. In particular, radiomic analysis of [^18^F]FDG PET imaging has been investigated in multiple settings of BC [15]. A large number of papers extracted radiomic features to predict molecular subtypes and histological features of BC at diagnosis, in a modern concept of “digital biopsy” [19]. In other experiences, the radiomic features of axillary lymph nodes were extracted and used to discriminate reactive from metastatic nodes [20]. Weber and colleagues [21] tested radiomics to increase the accuracy of PET imaging to predict the prognosis of BC patients. Moreover, a few papers demonstrated encouraging results by using radiomic analysis extracted by [^18^F]FDG PET to predict the response after NAC [22, 23].

Aim of this study is to build machine learning (ML) models able to predict pCR after NAC in BC patients based on conventional and radiomic signatures extracted from baseline [^18^F]FDG PET/CT.

## Material and methods

### Study population

This is a retrospective monocenter study involving newly diagnosed BC patients treated with NAC and studied with baseline [^18^F]FDG PET/CT between February 2015 and November 2023 at the Sant’Anna University Hospital of Ferrara (Italy).

The inclusion criteria of the study were: (a) age > 18 years, (b) histologically proven diagnosis of BC; (c) baseline [^18^F]FDG PET/CT performed prior to the first cycle of NAC; (d) history of NAC; (e) availability of histopathology and follow-up survival data.

The exclusion criteria were: (a) concomitant diagnosis of other oncological diseases; (b) patients with a previous diagnosis of BC who already received oncological treatment; (c) patients with evidence of distant metastases at baseline [^18^F]FDG PET/CT (d) patients who did not complete the proposed scheme of NAC; (e) patients who did not receive surgery after NAC.

Available clinical-pathological data were collected, including age, histology, grade, Estrogen Receptor (ER) expression, Progesterone Receptor (PR) expression, Ki-67 index, HER-2 expression, BRCA mutational status. The patients were classified according to the St. Gallen consensus [24] into 5 molecular subtypes subgroups: (1) Luminal A (ER-positive and/or PR-positive and HER-2 negative; Ki67 < 20%); (2) Luminal B (ER-positive and/or PR-positive and HER-2 negative; Ki67 ≥ 20%); (3) Luminal B + HER-2 (ER-positive and/or PR-positive and HER-2 positive; Ki67 ≥ 20%); (4) HER-2 enriched BC (without hormonal receptors expression and HER-2-positive); (5) TNBC (without expression of both hormonal receptors and HER-2 protein).

The study was performed according to the Declaration of Helsinki, Good Clinical Practice, and local ethical regulations. The local ethical committee approved the study (protocol number: 402/2024/Oss/AOUFE) and patients’ written informed consent was collected.

### NAC, surgery and histology

The types of NAC proposed were: (a) Epirubicin and Cyclophosphamide (EC) + Taxol; (b) EC + Taxol + Trastuzumab; (c) Taxol + Trastuzumab; (d) EC + Taxol + Carboplatin. The selection of the NAC scheme was performed according to the subtype of BC and observing the most updated guidelines at the time of the therapeutic decision.

All patients underwent breast surgery (breast-conservative and/or total mastectomy) and axillary staging (lymph node dissection and/or sentinel lymph node radioguided technique) after completing NAC.

At histological analysis after surgery, pCR was considered the main outcome after NAC, according to the Sataloff criteria [25] and defined as the complete absence of residual invasive tumor cells on microscopy, both in the breast and in the collected lymph nodes. Patients were divided as non-pCR or pCR, according to the absence (Sataloff Grades B, C and D), or evidence of pCR (Sataloff Grade A), respectively.

### *[*^*18*^*F]FDG PET/CT imaging*

PET/CT scans were performed according to a standardized protocol detailed in a previous paper ([14].

**Fig. 1:**
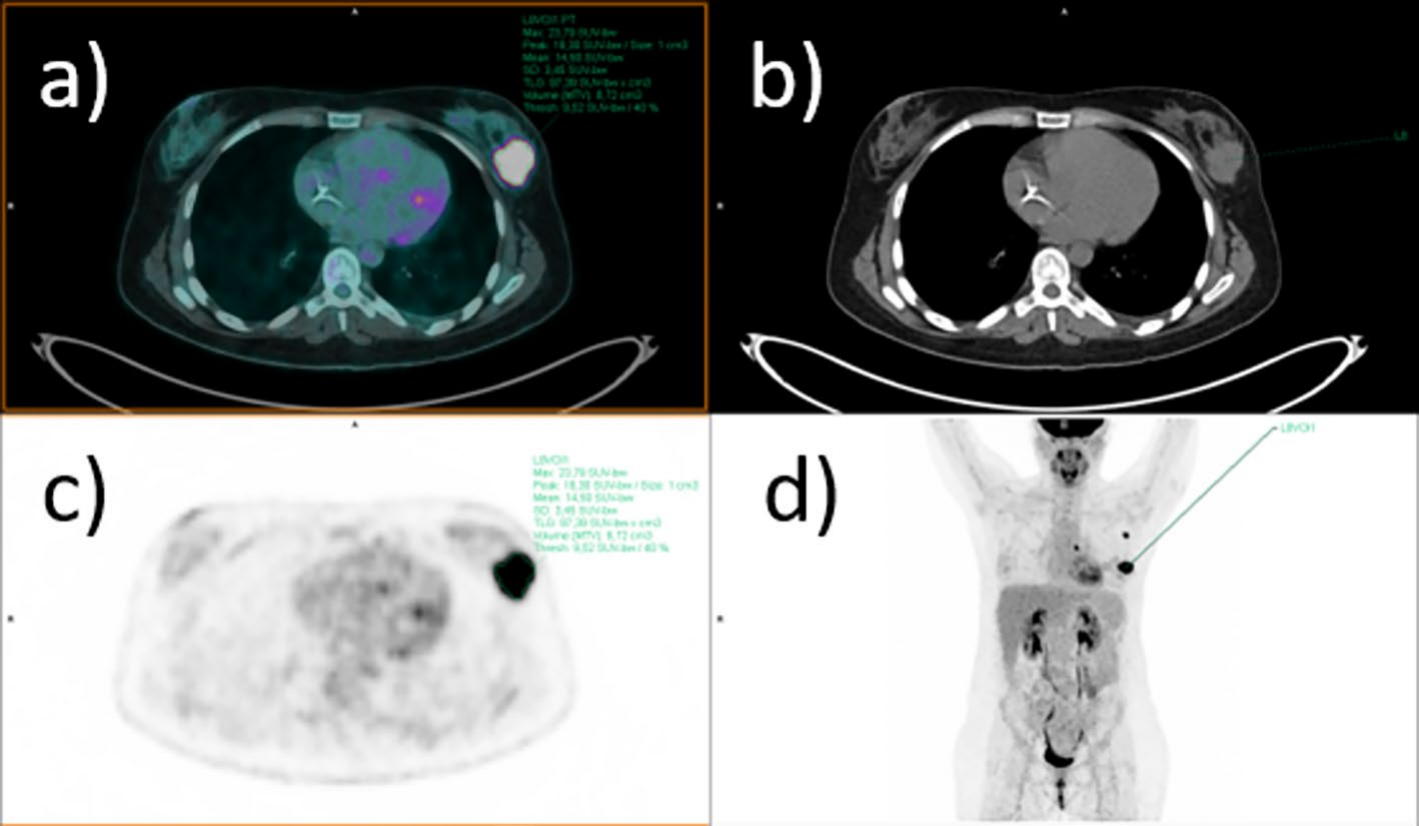
Baseline [^18^F]FDG PET/CT axial fused (**a**), CT (**b**), PET images (**c**) and MIP (maximum intensity projection) (**d**) of a 31 years-old women affected by TNBC. The exam shows focal [^18^F]FDG uptake (SUVmax = 23.8) in correspondence of the primary BC, as well as in a left axillary and in a homolateral internal mammary lymph nodes. The patient’s stage is therefore cT2;cN3. The patient achieved pCR after NAC

The analysis of [^18^F]FDG PET/CT was performed by two experienced nuclear medicine physicians on a syngo.via workstation (Siemens Healthineers, Enlargen, Germany), as shown in Fig. 1. The primary tumor and, when applicable, the pathological regional lymph nodes were identified on the PET images. Circular regions of interest (ROIs) were manually drawn and automatically converted in 3D volumes of interest (VOIs) by the software. The most common semiquantitative parameters were collected, including maximum and mean Standardized Uptake Value (SUVmax and SUVmean, respectively), metabolic tumor volume (MTV) and total lesion glycolysis (TLG). A 40% SUVmax thresholding was used to calculate volumetric parameters.

### Imaging postprocessing and features extraction

**Fig. 2:**
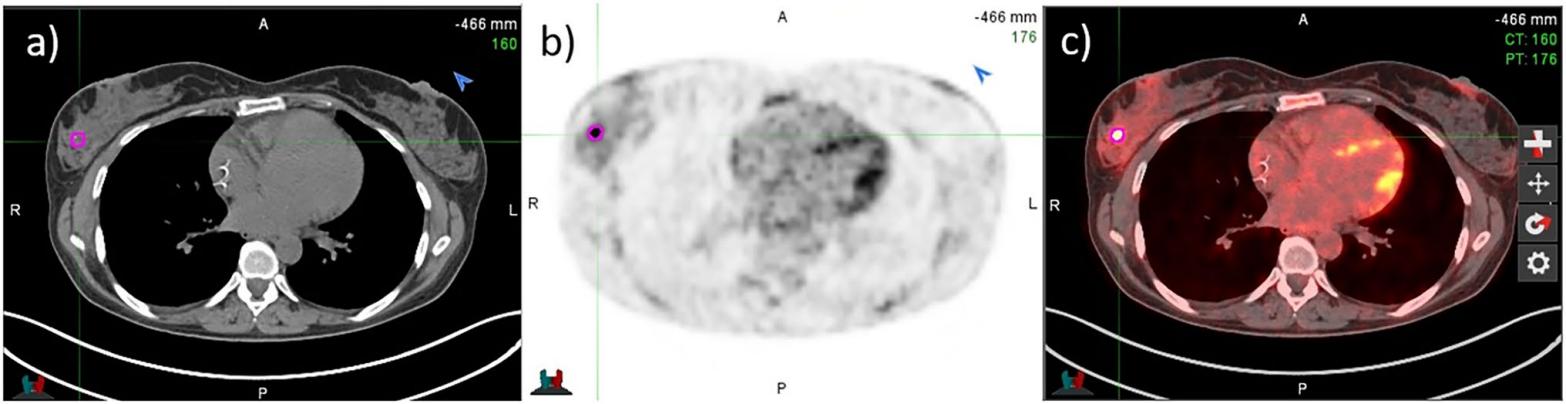
Manual segmentation of the VOI on transaxial CT (**a**), PET (**b**) and fused (**c**) images around the area of focal ^18^F-FDG uptake using MIM Maestro software

VOI segmentations of the primary tumor and the most significant lymph node metastasis (in terms of [^18^F]FDG uptake) were manually performed on [^18^F]FDG PET/CT images by two expert nuclear medicine physicians on MIM Maestro version 7.3.2 (MIM Software, Inc., Cleveland, OH), as shown in Fig. 2. Any discrepancies were resolved by consensus.

Quantitative radiomics features (RFts) were extracted from each VOIs on PET and only from those of the primary breast lesions on CT images. The process was performed separately using the Radiomics package and 3D Slicer image computing platform, according to IBSI standardization [26].

Before extracting the high-quantitative features, a discretization process with a fixed bin number of 25 was applied both for PET and CT images. Overall 121 radiomic features were extracted from each segmented VOI both on PET and CT images. Among those, respectively divided by classes, 14 RFts belong to original image and mask, 14 to Shape (3D) class, 18 to First Order intensity statistics, 24 to Gray Level Co-occurrence Matrix (GLCM), 16 to Gray Level Run Length Matrix (GLRLM), 16 to Gray Level Size Zone (GLSZM), 14 to Gray Level Dependence Matrix (GLDM) and 5 to Neighboring Gray Tone Difference Matrix (NGTDM). In addiction 744 textural RFts were extracted from wavelet decomposed VOIs.

### Statistical analysis and model construction

The correlation between clinical parameters, [^18^F]FDG PET/CT metabolic (SUVmax, SUVmean) and volumetric (MTV and TLG) parameters and the pCR/non-pCR group was investigated using Two-tailed Wilcoxon-Mann–Whitney U-type test (*p* < 0.05). A sub-analysis of semiquantitative parameters in each molecular subgroups was performed with Pearson Chi square test (*p* < *0.05*).

Before RFts reduction, The Synthetic Minority Over-Sampling Technique (SMOTE) strategy was used to balance the sample distribution of non-pCR and pCR into the dataset, obtaining a sample size of 78 lesions, 39 labeled non-pCR and 39 pCR. The cohort was then randomly divided into training set (70%) and internal validation set (30%). The training set was used to build a model for the prediction of pCR response after NAC.

A filter feature selection algorithm using a handcrafted python script was implemented to identify robust RFTs. Prior to the feature selection step, no data transformation or normalization was conducted. To evaluate the most robust, non-redundant and most reproducible RFts a *p* < 0.05 and a |r_s_|> 0.8 of the Wilcoxon-Mann-Witney U type test and Spearman Rank correlation coefficient respectively was considered. This analysis was independently performed for both CT-based and PET-based RFts datasets.

The bootstrap method and 10-Fold Cross Validation were used for the training of the ML Models. Four ML prediction models (PET Model_T, PET Model_T + N, CT Model and Clinical Model) were independently built. PET and CT Models were built using RFts selected and extracted from the corresponding images dataset (all VOIs for the PET Model_T + N; primary BC VOIs for PET Model_T and CT Model), whereas the Clinical Model was built using 8 clinical-pathological signatures (age, histology, grade, ER, PR, HER-2, Ki-67 and BRCA).

Orange data mining was used for training, test and validation of the different ML Models. For each ML Model, three different algorithms were trained: Random Forest (RF), Stochastic Gradient Descent (SGD) and Neural Network (NN).

Area under the ROC curve (AUC), Classification Accuracy (CA), Precision, Sensitivity, Specificity, True-Positive (TP) and True-Negative (TN) were calculated in the training and validation steps. This performance scores were used to evaluate the best algorithm for each ML Model.

## Results

### Study population and NAC response

Fifty-two females with newly diagnosed BC met the inclusion criteria. Median intercurrent time between the baseline [^18^F]FDG PET/CT and the first day of NAC was 9 (2–20) days. Overall, 72 lesions detected by [^18^F]FDG PET/CT were considered for the analysis. Among those, all the 52 primary tumors showed increased [^18^F]FDG uptake (mean SUVmax = 14.10 ± 12.22). Moreover, 20 (38.5%) patients showed lymph node metastasis. Most of the patients with lymph node metastasis were affected by TNBC (n = 6; 30%) and HER-2 positive BC (n = 9; 45%), while only 5 patients (20%) with Luminal A or Luminal B BC had nodal involvement.

Overall, pCR after NAC was achieved in 23 patients (44.2%). Among the different BC subtypes, pCR was more frequently reached in HER-2 enriched BC (n = 10; 91%) and TNBC (n = 8; 42%). Detailed characteristics of patients included are shown in Table 1.

**Table 1:** Characteristics of the study population

Variables	Non-pCR	pCR	Total
Number of patients	29 (56%)	23(44%)	52 (100%)
AGE [years]			
Mean ± St.dev	56 ± 11	53 ± 13	54 ± 12
Range	36–77	32–76	32–77
Histology			
IDC	25 (86%)	21 (91%)	46 (88%)
ILC	1 (3%)	0 (0%)	1 (2%)
Mixed	1 (3%)	1 (4%)	2 (4%)
Others	2 (6%)	1 (4%)	2 (6%)
Grade			
G1	0 (0%)	0 (0%)	0 (0%)
G2	9 (31%)	5 (22%)	14 (27%)
G3	20 (69%)	18 (78%)	38 (73%)
HER-2 expression			
Yes	12 (41%)	12 (52%)	24 (46%)
No	17 (59%)	11 (48%)	28 (54%)
Ki-67 median (range)	46% (11%-85%)	44% (12%-90%)	45% (11%-90%)
Subtype			
Luminal A	1 (3%)	1 (4%)	2(4%)
Luminal B	4 (14%)	2 (9%)	6 (12%)
Luminal B + HER-2	12 (41%)	2 (9%)	14 (27%)
HER-2 + non Luminal	1 (3%)	10 (43%)	11 (21%)
TNBC	11 (38%)	8 (35%)	19 (37%)
BRCA			
Yes	0 (0%)	3 (13%)	3 (6%)
No	9(31%)	11 (48%)	20 (38%)
NA	20 (69%)	9 (39%)	29 (56%)
Breast PET			
Yes	29	23 (0%)	52 (100%)
No	0 (0%)	0 (0%)	0 (0%)
Axillary LN PET			
Yes	10	10	20 (38.5%)
No	19	13	32 (61.5%)

Pathological complete response (pCR); Standard deviation (St. dev.); Invasive ductal carcinoma (IDC); Invasive lobular carcinoma (ILC); Human epidermal growth factor receptor 2 (HER-2); Triple-negative breast cancer (TNBC); Positron emission tomography (PET); Lymph nodes (LN)

### *[*^*18*^*F]FDG PET/CT semiquantitative parameters of the primary tumor*

The results of the analysis of the semiquantitative parameters extracted from the primary BC to discriminate pCR vs non-pCR are shown in Table 2. Metabolic parameters (SUVmax and SUVmean) of the primary tumor resulted non-significant in discriminating between pCR and non-pCR groups. Conversely, patients who did not achieve pCR after NAC had significantly higher mean values of the volumetric parameters relative to the primary tumor (*p* = 0.004 and *p* = 0.001 for MTV and TLG, respectively). A subanalysis performed by tumor subtype did not provide significant results.

**Table 2:** Analysis of the [^18^F]FDG PET/CT semiquantitative parameters extracted from the primary BC and subtypes subgroups for predicting pCR after NAC

	Non-pCR	pCR	Total	*p *value
SUVmax				0.670
Mean ± St.dev	16.77 ± 14.82	10.74 ± 6.75	14.10 ± 12.22	
Range	2.5–71.7	2.7–26.3	2.5–71.7	
SUVmean				0.830
Mean ± St.dev	10.34 ± 8.95	6.49 ± 4.18	8.64 ± 7.43	
Range	1.4–41.7	1.6–15.2	1.4–41.7	
MTV				0.004
Mean ± St.dev	8.20 ± 10.92	4.40 ± 6.25	6.52 ± 9.27	
Range	1–44	0.6–24.6	0.6–44	
TLG				0.001
Mean ± St.dev	96.92 ± 199.63	25.20 ± 31.08	65.20 ± 153.59	
Range	4.6–994.9	2.4–106.6	2.4–994.9	

Positron emission tomography/computed tomography (PET/CT); Breast cancer (BC); Pathological complete response (pCR); NeoAdjuvant chemotherapy (NAC); Standardized uptake value (SUV); Standard deviation (St. dev); Metabolic tumor volume (MTV); Total lesion glycolysis (TLG)

### Radiomic analysis and model building

Among the 854 RFts extracted from original and filtered images, significant statistical differences between non-pCR and pCR groups were found for a total of 12, 46 and 141 robust RFts for CT, PET_T and PET_T + N image datasets, respectively.

In the CT dataset 12 RFts resulted robust. Among them, 4 belong to texture class (GLDM and GLSZM and GLRLM) and 8 to the texture wavelet-based class.

In the PET_T + N dataset, 141 robust RFts were selected (5 belonging to the shape class, 12 to the first and second order GLCM and 124 to the textural wavelet-based class) along with MTV and TLG. On the other hand, in the PET_T dataset, 46 RFts were selected (1 belonging to the SHAPE class, 1 to the GLDM class and 44 to the texture wavelet-based class). The full list of selected RFts is shown in Supplementary material (Tables S1, S2, and S3).

The performances of the 10-Fold CV, in all of the 3 learners, showed AUC > 0.63 and CA > 0.62 in the Clinical Model; AUC > 0.64 and CA > 0.73 in CT Model, AUC > 0.82 and CA > 0.76 in the PET Model_T. In the training phase, the model that showed the better performances was the PET Model_T + N with AUC > 0.89 and CA > 0.84. Table 3 displays the performances of the three learners in 10-FoldCV across the four different models.

**Table 3:** Performances scores in 10-Fold CV for each ML Model

ML model		AUC	CA	Precision	Sensitivity	Specificity
Clinical Model	NN	0.63	0.62	0.62	0.62	0.70
	RF	0.76	0.65	0.64	0.65	0.66
	SGD	0.73	0.65	0.65	0.65	0.78
CT Model	NN	0.82	0.88	0.88	0.88	0.85
	RF	0.89	0.88	0.88	0.88	0.82
	SGD	0.64	0.73	0.76	0.73	0.56
PET Model_T	NN	0.90	0.90	0.90	0.90	0.79
	RF	0.89	0.88	0.88	0.88	0.66
	SGD	0.82	0.76	0.75	0.76	0.81
PET Model_T + N	NN	0.91	0.91	0.91	0.91	0.88
	RF	0.94	0.87	0.87	0.87	0.83
	SGD	0.89	0.84	0.84	0.84	0.81

Machine learning (ML); Area under the curve (AUC); Neural network (NN); Random forest (RF); Stochastic gradient DEscent (SGD); Computed tomography (CT); Positron emission tomography (PET)

The classification performances in the validation step are shown in Table 4. Overall, PET Models showed better results (AUC, CA, precision, sensitivity, specificity, TP and TN all above 0.61) using all the three algorithms. The best performances were obtained by RF into the PET Model_T + N (AUC = 0.83; CA = 0.74; precision = 0.80, sensitivity = 0.76, specificity = 0.76, TP = 0.60; TN = 0.61). Also, PET Model_T showed better performances than the CT Model, with the best classifier (RF) obtaining AUC = 0.76 and CA = 0.70 vs AUC = 0.44 and CA = 0.47, respectively.

**Table 4:** Performances scores in the validation step for each ML Model

ML model		AUC	CA	Precision	Sensitivity	Specificity	TP (%)	TN (%)
Clinical Model	NN	0.59	0.73	0.82	0.73	0.57	60.00	54.50
	RF	0.68	0.67	0.63	0.67	0.57	57.90	55.60
	SGD	0.57	0.67	0.69	0.67	0.48	22.20	39.30
CT Model_T	NN	0.59	0.35	0.43	0.35	0.50	43.00	40.00
	RF	0.44	0.47	0.62	0.47	0.64	75.00	38.50
	SGD	0.56	0.47	0.79	0.47	0.71	50.00	30.80
PET Model_T	NN	0.79	0.65	0.64	0.65	0.65	66.70	72.50
	RF	0.76	0.70	0.71	0.70	0.71	75.00	66.70
	SGD	0.74	0.64	0.64	0.64	0.64	66.70	62.50
PET Model_T + N	NN	0.78	0.65	0.66	0.65	0.65	61.50	70.00
	RF	0.83	0.74	0.80	0.74	0.76	77.80	71.40
	SGD	0.72	0.61	0.61	0.61	0.63	58.30	63.60

Machine learning (ML); Area under the curve (AUC); Classification accuracy (CA); True-positive (TP); True-negative (TN); Neural network (NN); Random forest (RF); Stochastic gradient DEscent (SGD); Computed tomography (CT); Positron emission tomography (PET)

The Clinical Model, trained without radiomic data, showed lower ability to predict the response after NAC in BC patients (TP = 57.9% and TN = 55.60%).

**Fig. 3:**
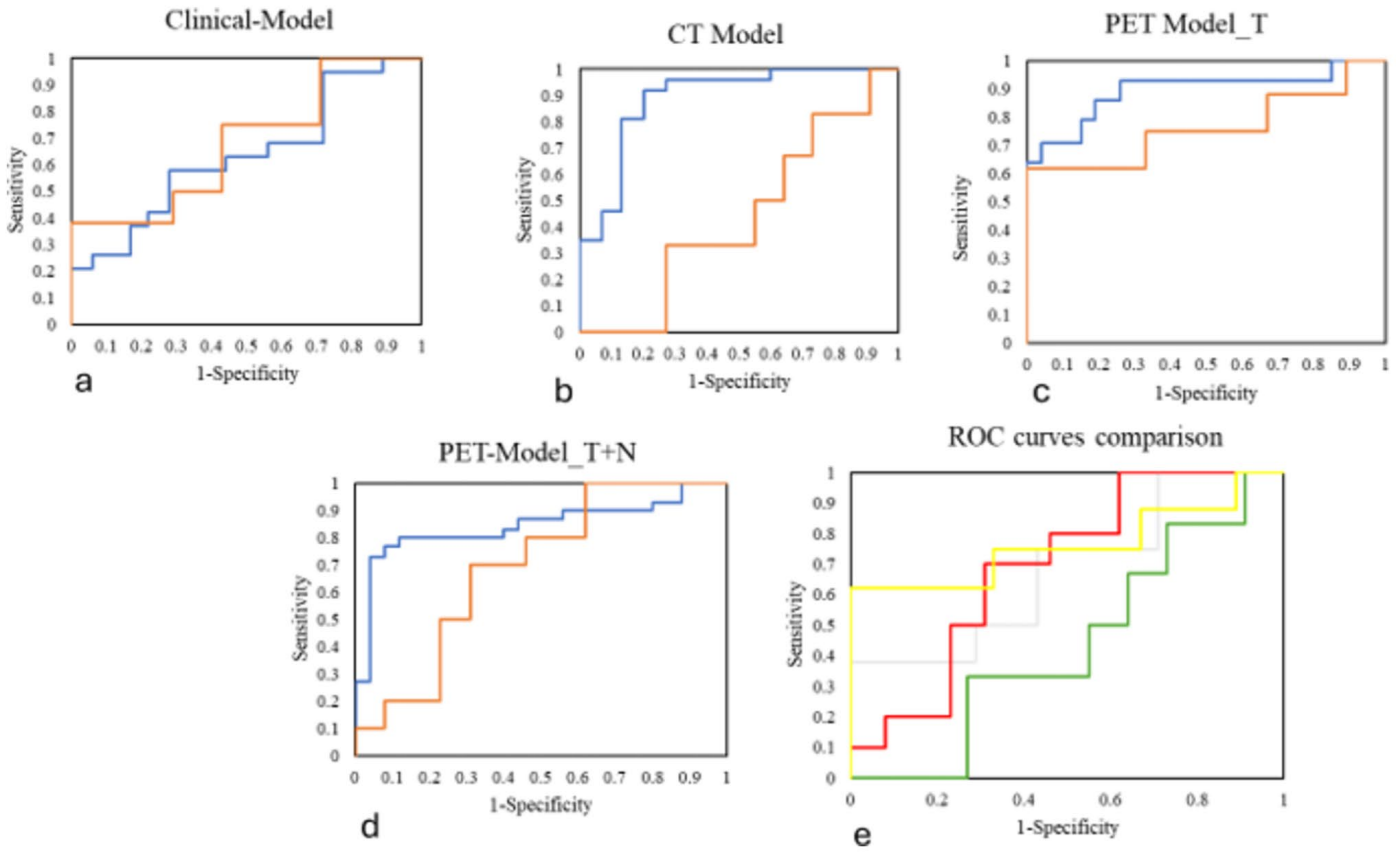
ROC curves of the RF learner relative to the Clinical Model (**a**), CT Model (**b**), PET Model_T (**c**) and PET Model_T + N (**d**). Blue line: 10-Fold CV, orange line: validation. (**e**) Comparison of the ROC curves of the RF learner in the Clinical Model (gray), CT Model (green), PET Model_T (yellow) and PET Model_T + N (red)

ROC curves of the best ML Models are graphically shown in Fig. 3.

**Fig. 4:**
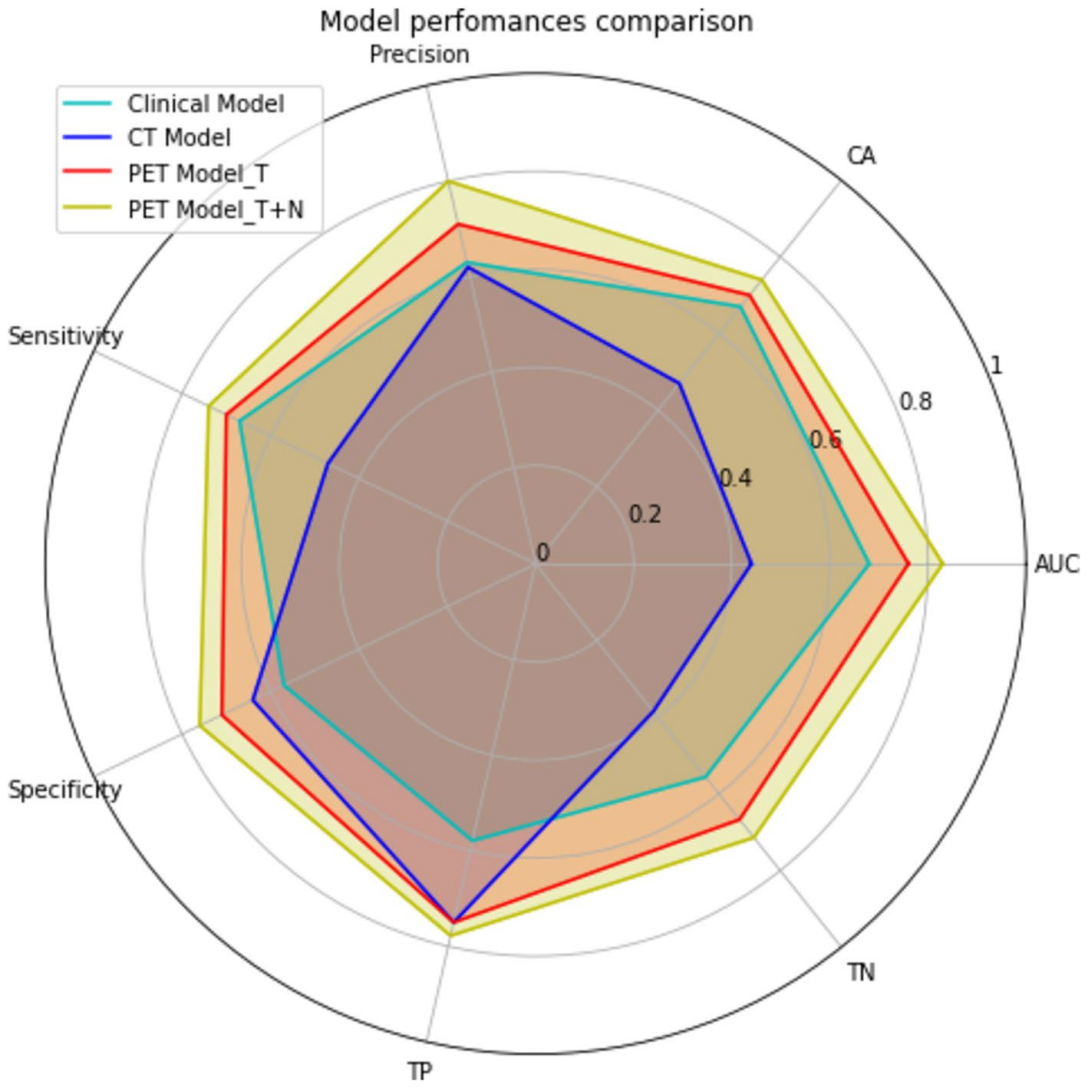
Comparison of random forest learner performances scores of the different ML Models. The figure highlights the greater potential of the PET Model_T + N

In addiction a radial plot was built to realize a visual comparison of the performance scores of the learners (NN, RF and SGD) for each of the 4 ML models trained (Fig. S1). Figure 4 represents a graphical comparison of the performances of RF, that resulted the best learner in terms of performances among the four models.

**Fig. 5:**
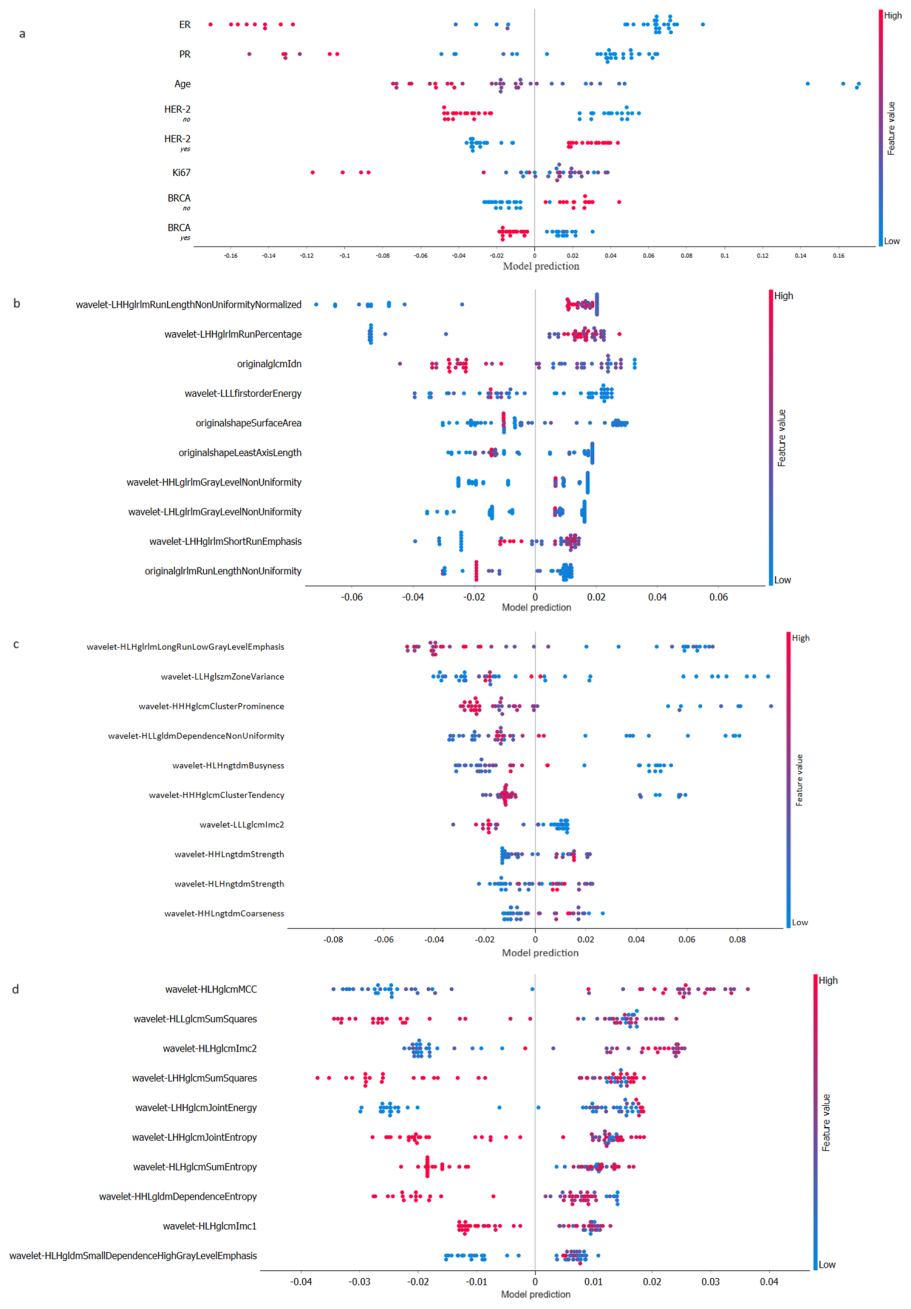
SHAP features relevance in the RF of the 4 ML models. The graphed features are all of the Clinical Model (**a**) and the best ten for the CT Model (**b**), the PET Model_T (**c**) and the PET Model_T + N (**d**). Features are ranked in order of their importance. Each dot represents a single patient’s prediction. Colors indicate the magnitude of the feature value in each row for a given patient. The x-axis indicates the model’s tendency for the prediction of pCR or non-pCR due to a given feature value in a given patient

Finally, a shapely additive explanation (SHAP) analysis was graphed to demonstrate how features within the RF pCR classifier impacted the model output, in each of the 4 models (Fig. 5).

## Discussion

The prognostic relevance of pCR after NAC in BC patients has been widely investigated in literature in the last decades [4]. We interrogated PubMed on June 20, 2024 with the following keywords: “pcr and breast cancer and nac”. The results displayed passed from < 5/year in the early 2000s to more than 150/year in 2022 and 2023. The impressive increased number of reports in literature in recent years testifies the great research interest in this parameter. However, pCR is assessed after surgery, thus reducing the therapeutic adjustment only to the initiation of adjuvant treatment in patients with evidence of residual disease. The accurate early prediction of pCR after NAC would be highly relevant to tailor presurgical treatment in BC patients, enabling treatment modulation and potentially improving response rate. For this task, we firmly believe imaging is the main option. Breast MRI and [^18^F]FDG PET/CT have been investigated for this purpose [6, 27]. However, post-NAC scans may present false positive or false negative findings [28–30].

In our previous experience we analyzed volumetric parameters at baseline [^18^F]FDG PET/CT and reported that higher values of MTV and TLG of the primary tumor were correlated with residual disease after NAC in Luminal B + HER-2 BC patients [14]. In the current study we confirmed that patients showing higher values of volumetric parameters relative to the primary tumor at baseline [^18^F]FDG PET/CT are unlikely to achieve pCR after NAC, regardless of the tumor subtype. In a previous study Evangelista et al. [31] found that higher values of volumetric parameters at baseline [^18^F]FDG PET/CT were also associated to shorter PFS in BC patients treated with NAC. Taken together, these results highlight the prognostic relevance of the volumetric extent of the metabolically active burden of disease at [^18^F]FDG PET/CT performed before starting NAC in BC patients. However, the value of volumetric parameters is very heterogeneous and tomograph-dependent, thus precluding the possibility to define universal cutoff values that may be used in daily clinical practice.

To overcome the limits of conventional imaging analysis it could be combined with modern radiomic analysis, which enables the collection of hundreds features otherwise hidden to the human eye [32]. Several papers explored the potential of radiomics analysis applied to breast ultrasound and MRI to build AI models able to predict pCR after NAC, with very promising results [33–35]. Similarly, a few papers tried the same workflow, but on [^18^F]FDG PET/CT or PET/MRI [15, 36–38]. Lee et al. [39] reported suboptimal performances of their models built on clinical-pathological and radiomic features. More encouraging results were reported by Umutlu et al. [22]. However, the authors combined radiomic features of the primary breast tumor, extracted from both PET and MRI images. Recently, Lim et al. [40] developed and externally validated a model built on [^18^F]FDG PET/CT radiomic features and significant clinical variables that resulted able to accurately predict pCR after NAC (AUC = 0.729 at external validation). However, once again the model was built using only the radiomic features extracted from the primary breast tumors. In this study, we built a PET Model trained on robust radiomic features extracted from both the primary BC and the reference lymph node lesion, namely PET Model_T + N. The rationale behind this choice is that a pathological uptake at [^18^F]FDG PET reflects the metabolic pattern of tumoral cells within the target lesion. Therefore, we hypothesized that the extraction of robust radiomic features also from the most relevant lymph node metastasis would provide useful additional information to the model. Our results look promising as we managed to build ML models able to accurately predict pCR after NAC. Noteworthy, PET Model_T + N outperformed PET Model_T (AUC = 0.83, CA = 0.74, vs AUC = 0.76, CA = 0.70, respectively), confirming our hypothesis that including the RFts extracted from the reference lymph node may improve the performances of the ML model. These preliminary results look promising and should be considered for external validation in upcoming experiences with larger sample size.

As expected, both PET models showed markedly better performances than the CT Model. The CT scan co-registered with PET imaging was a low-dose CT without contrast enhancement (ce), thus suboptimal source of radiomic data in comparison to a diagnostic ceCT. Indeed, focusing on the PET Model_T and the CT Model (both trained only with RFts extracted from the primary breast tumor in the two respective imaging datasets), the first showed better performances probably due to the statistical fluctuation determined by the lack of robust RFts in the CT dataset, producing an overfitting error. Overfitting occurs when a model learns not only the underlying patterns in the training data but also the noise and random fluctuations. As a consequence, the model performs well on the training data but poorly and with low stability on new, unseen data. This is the case of the CT Model, that shows good results in 10-Fold Cross Validation (AUC = 0.89; CA = 0.88) that considerably decrease at validation step (AUC = 0.44; CA = 0.47). Conversely, in PET models the radiomics features represent a large set of quantitative parameters able to discriminate the population and overcoming overfitting at internal validation. However, external validation is needed in future experiences to confirm our promising preliminary results.

Our strategy to include lymph node metastasis only in the radiomic analysis of PET imaging was considered because the metabolic pattern of the tumoral cells within a lesion might be scarcely influenced by the metabolism of the background tissue, both in breast and lymph nodes. Conversely, CT is a morphological imaging and the radiomic features may be influenced by the different physiological density of the tissue considered, unless there is evidence of macroscopic neoplastic subversion of the tissue itself. Therefore, we did not include lymph node CT VOIs into the radiomic analysis.

Considering the clinical Model, the performances resulted suboptimal, as expected considering that clinical-pathological signatures did not result statistically significant to discriminate pCR Vs non-pCR BC in our cohort. Moreover, the relatively small sample size and the unbalanced population did not allow adequate training of the Clinical Model.

A few limitations of our study should be mentioned. Firstly, the retrospective design, the relatively small sample and the lack of external validation represent flaws of the current study that could be overcome in future experiences. Although HER-2 enriched BC are associated to higher response rates than other BC subtypes, HER-2 enriched BC showed a very high rate of pCR after NAC (91%) in our population study, which is higher than that expected according to literature (65%) [41]. However, the ML models presented in this study were trained considering a mixed cohort of BC subtypes. Therefore, the lack of non-responders in the HER-2 enriched group should not have a relevant impact on the reliability of the models presented.

In conclusion, radiomic analysis of baseline [^18^F]FDG PET/CT has the potential to contribute to the prediction of pCR after NAC. A validation of our model on external cohort may contribute to personalize the treatment of BC patients unlikely to achieve complete response after therapy.

## Supplementary Information

Below is the link to the electronic supplementary material.

Supplementary file1 (DOCX 19 KB)

Supplementary file2 (DOCX 26 KB)

Supplementary file3 (DOCX 42 KB)

Supplementary file4 (DOCX 329 KB)
