# Supplementary material for: 18F-FDG PET/CT radiomic analysis and artificial intelligence to predict pathological complete response after neoadjuvant chemotherapy in breast cancer patients
Source: Radiol Med. 2025 Jan 28;130(4):543–54. doi: 10.1007/s11547-025-01958-4 (PMC12008070; doi:10.1007/s11547-025-01958-4)
Supplement: Supplementary file 1 — Supplementary file1 (DOCX 19 KB) [file 11547_2025_1958_MOESM1_ESM.docx]

**Table1S: Robust CT derived radiomic features.**

| Filter | Class | Radiomic feature | pCR1 | pCR0 | Tot | p | \|rs\| |
| --- | --- | --- | --- | --- | --- | --- | --- |
| original | gldm | SmallDependenceLowGrayLevelEmphasis | 4.76E-3±2.71E-3 | 3.45E-3±1.9E-3 | 4.11E-3±2.42E-3 | 0.043 | 0.978 |
| original | glrlm | RunEntropy | 3.83E+0±3.21E-1 | 4.02E+0±2.73E-1 | 3.92E+0±3.11E-1 | 0.032 | 0.881 |
| original | glszm | LargeAreaEmphasis | 2.49E+4±4.31E+4 | 9.16E+4±1.84E+5 | 5.83E+4±1.37E+5 | 0.043 | 0.887 |
| original | glszm | ZoneVariance | 2.43E+4±4.28E+4 | 9.05E+4±1.84E+5 | 5.74E+4±1.36E+5 | 0.042 | 0.827 |
| wavelet-LLH | gldm | GrayLevelNonUniformity | 1.11E+3±1.71E+3 | 2.54E+3±3.71E+3 | 1.83E+3±2.96E+3 | 0.027 | 0.845 |
| wavelet-LHH | glrlm | RunVariance | 1.2E+0±2.9E-1 | 1.39E+0±3.38E-1 | 1.3E+0±3.26E-1 | 0.017 | 0.847 |
| wavelet-HLH | glrlm | RunVariance | 1.17E+0±2.73E-1 | 1.32E+0±3.3E-1 | 1.25E+0±3.09E-1 | 0.039 | 0.881 |
| wavelet-HHL | glrlm | RunVariance | 1.05E+0±2.44E-1 | 1.21E+0±3.04E-1 | 1.13E+0±2.85E-1 | 0.021 | 0.811 |
| wavelet-HHL | glszm | ZoneVariance | 3.22E+5±8.64E+5 | 3.37E+6±1.08E+7 | 1.85E+6±7.76E+6 | 0.04 | 0.964 |
| wavelet-HHH | glrlm | RunVariance | 9.97E-1±2.28E-1 | 1.15E+0±2.53E-1 | 1.07E+0±2.5E-1 | 0.019 | 0.998 |
| wavelet-LLL | gldm | SmallDependenceLowGrayLevelEmphasis | 3.69E-3±2.99E-3 | 2.37E-3±1.88E-3 | 3.03E-3±2.57E-3 | 0.009 | 0.978 |
| wavelet-LLL | glszm | SmallAreaLowGrayLevelEmphasis | 1.56E-2±1.4E-2 | 1.07E-2±6.95E-3 | 1.32E-2±1.12E-2 | 0.036 | 0.863 |
| Computed Tomography (CT); Pathological Complete Response (pCR); Spearman’s rank correlation coefficient (r_S_) | | | | | | | |
