# Supplementary material for: 18F-FDG PET/CT radiomic analysis and artificial intelligence to predict pathological complete response after neoadjuvant chemotherapy in breast cancer patients
Source: Radiol Med. 2025 Jan 28;130(4):543–54. doi: 10.1007/s11547-025-01958-4 (PMC12008070; doi:10.1007/s11547-025-01958-4)
Supplement: Supplementary file 2 — Supplementary file2 (DOCX 26 KB) [file 11547_2025_1958_MOESM2_ESM.docx]

**Table2S: Robust PET_T derived radiomic features.**

| Filter | Class | Radiomic feature | pCR1 | pCR0 | Tot | p | \|r_s_\| |
| --- | --- | --- | --- | --- | --- | --- | --- |
| original | shape | VoxelVolume | 6.93E+3±9.94E+3 | 1.397E+4±1.916E+4 | 1.05E+4±1.55E+4 | 0.049 | 0.806 |
| original | gldm | DependenceNonUniformity | 1.E+1±1.23E+1 | 2.376E+1±3.983E+1 | 1.69E+1±3.E+1 | 0.027 | 0.921 |
| wavelet-LLH | glszm | SizeZoneNonUniformityNormalized | 4.87E-1±2.42E-1 | 3.529E-1±1.043E-1 | 4.2E-1±1.96E-1 | 0.041 | 0.97 |
| wavelet-LLH | glszm | ZoneVariance | 3.02E+3±1.13E+4 | 1.224E+4±3.138E+4 | 7.63E+3±2.38E+4 | 0.015 | 0.823 |
| wavelet-LHL | glszm | ZoneEntropy | 1.2E+0±3.87E-1 | 1.476E+0±5.62E-1 | 1.34E+0±4.98E-1 | 0.045 | 0.944 |
| wavelet-LHH | firstorder | orderMedian | 6.71E-3±5.72E-2 | -2.125E-2±4.606E-2 | -7.27E-3±5.34E-2 | 0.027 | 0.899 |
| wavelet-LHH | glszm | ZonePercentage | 5.59E-2±7.59E-2 | 3.518E-2±5.76E-2 | 4.55E-2±6.76E-2 | 0.038 | 0.969 |
| wavelet-LHH | ngtdm | Coarseness | 1.09E-1±1.52E-1 | 6.576E-2±1.016E-1 | 8.74E-2±1.3E-1 | 0.031 | 0.867 |
| wavelet-LHH | ngtdm | Strength | 1.08E-1±1.48E-1 | 6.572E-2±1.016E-1 | 8.67E-2±1.28E-1 | 0.031 | 0.841 |
| wavelet-HLL | gldm | DependenceEntropy | 4.06E+0±9.2E-1 | 4.373E+0±9.693E-1 | 4.22E+0±9.5E-1 | 0.026 | 0.897 |
| wavelet-HLL | gldm | DependenceNonUniformity | 1.08E+1±1.16E+1 | 1.959E+1±2.435E+1 | 1.52E+1±1.94E+1 | 0.031 | 0.821 |
| wavelet-HLH | gldm | DependenceNonUniformity | 1.26E+1±1.51E+1 | 2.361E+1±3.115E+1 | 1.81E+1±2.49E+1 | 0.043 | 0.851 |
| wavelet-HLH | gldm | GrayLevelNonUniformity | 7.02E+1±9.97E+1 | 1.41E+2±1.927E+2 | 1.06E+2±1.56E+2 | 0.046 | 0.873 |
| wavelet-HLH | glrlm | LongRunLowGrayLevelEmphasis | 1.57E+0±4.E-1 | 2.005E+0±6.084E-1 | 1.79E+0±5.55E-1 | 0.004 | 0.893 |
| wavelet-HLH | ngtdm | Busyness | 3.38E+1±4.97E+1 | 7.295E+1±1.012E+2 | 5.34E+1±8.14E+1 | 0.027 | 0.905 |
| wavelet-HLH | ngtdm | Strength | 1.01E-1±1.26E-1 | 7.992E-2±1.475E-1 | 9.06E-2±1.37E-1 | 0.045 | 0.823 |
| wavelet-HHL | glrlm | GrayLevelNonUniformity | 4.7E+1±6.31E+1 | 8.766E+1±1.116E+2 | 6.73E+1±9.22E+1 | 0.047 | 0.819 |
| wavelet-HHL | glszm | SmallAreaEmphasis | 9.75E-2±1.44E-1 | 8.985E-2±1.977E-1 | 9.37E-2±1.71E-1 | 0.045 | 0.972 |
| wavelet-HHL | ngtdm | Coarseness | 1.18E-1±1.75E-1 | 8.265E-2±1.867E-1 | 1.E-1±1.8E-1 | 0.025 | 0.98 |
| wavelet-HHL | ngtdm | Strength | 1.15E-1±1.66E-1 | 8.295E-2±1.871E-1 | 9.88E-2±1.76E-1 | 0.026 | 0.982 |
| wavelet-HHH | glcm | ClusterProminence | 4.49E-1±9.96E-2 | 4.719E-1±9.113E-2 | 4.6E-1±9.54E-2 | 0.022 | 0.899 |
| wavelet-HHH | glcm | ClusterTendency | 4.56E-1±8.09E-2 | 4.738E-1±8.422E-2 | 4.65E-1±8.24E-2 | 0.017 | 0.951 |
| wavelet-HHH | glrlm | RunLengthNonUniformity | 5.03E+1±6.15E+1 | 8.958E+1±1.089E+2 | 6.99E+1±8.99E+1 | 0.048 | 0.893 |
| wavelet-HHH | ngtdm | Coarseness | 9.73E-2±1.35E-1 | 6.643E-2±1.187E-1 | 8.18E-2±1.27E-1 | 0.029 | 0.815 |
| wavelet-HHH | ngtdm | Strength | 9.67E-2±1.33E-1 | 6.644E-2±1.187E-1 | 8.16E-2±1.26E-1 | 0.029 | 0.815 |
| wavelet-LLL | firstorder | Entropy | 2.75E-1±4.4E-1 | 6.532E-1±7.04E-1 | 4.64E-1±6.12E-1 | 0.013 | 0.942 |
| wavelet-LLL | firstorder | Uniformity | 8.76E-1±1.98E-1 | 7.359E-1±2.655E-1 | 8.06E-1±2.43E-1 | 0.012 | 0.896 |
| wavelet-LLL | glcm | Autocorrelation | 1.39E+0±7.1E-1 | 2.536E+0±2.78E+0 | 1.96E+0±2.09E+0 | 0.01 | 0.919 |
| wavelet-LLL | glcm | ClusterProminence | 2.94E-1±6.19E-1 | 6.665E+0±2.256E+1 | 3.48E+0±1.61E+1 | 0.009 | 0.932 |
| wavelet-LLL | glcm | Correlation | 7.58E-1±3.75E-1 | 5.042E-1±3.698E-1 | 6.31E-1±3.91E-1 | 0.018 | 0.936 |
| wavelet-LLL | glcm | Imc2 | 8.3E-2±1.44E-1 | 2.264E-1±2.252E-1 | 1.55E-1±2.01E-1 | 0.003 | 0.852 |
| wavelet-LLL | glcm | JointAverage | 1.15E+0±2.57E-1 | 1.44E+0±6.333E-1 | 1.29E+0±5.01E-1 | 0.01 | 0.919 |
| wavelet-LLL | glcm | SumAverage | 2.29E+0±5.06E-1 | 2.867E+0±1.25E+0 | 2.58E+0±9.89E-1 | 0.01 | 0.995 |
| wavelet-LLL | gldm | GrayLevelVariance | 6.83E-2±1.13E-1 | 2.763E-1±5.218E-1 | 1.72E-1±3.89E-1 | 0.013 | 0.985 |
| wavelet-LLL | gldm | HighGrayLevelEmphasis | 1.35E+0±6.45E-1 | 2.386E+0±2.471E+0 | 1.87E+0±1.86E+0 | 0.008 | 0.985 |
| wavelet-LLL | gldm | LowGrayLevelEmphasis | 9.18E-1±1.47E-1 | 7.977E-1±2.432E-1 | 8.58E-1±2.08E-1 | 0.009 | 0.985 |
| wavelet-LLL | glrlm | GrayLevelNonUniformityNormalized | 8.75E-1±2.E-1 | 7.187E-1±2.612E-1 | 7.97E-1±2.44E-1 | 0.009 | 0.959 |
| wavelet-LLL | glrlm | GrayLevelVariance | 7.21E-2±1.22E-1 | 2.841E-1±5.126E-1 | 1.78E-1±3.85E-1 | 0.008 | 0.912 |
| wavelet-LLL | glrlm | HighGrayLevelRunEmphasis | 1.31E+0±5.64E-1 | 2.294E+0±2.341E+0 | 1.8E+0±1.76E+0 | 0.008 | 0.959 |
| wavelet-LLL | glrlm | LowGrayLevelRunEmphasis | 9.28E-1±1.23E-1 | 8.112E-1±2.16E-1 | 8.7E-1±1.84E-1 | 0.008 | 0.985 |
| wavelet-LLL | glszm | HighGrayLevelZoneEmphasis | 1.64E+0±1.12E+0 | 2.996E+0±2.716E+0 | 2.32E+0±2.17E+0 | 0.006 | 0.991 |
| wavelet-LLL | glszm | LowGrayLevelZoneEmphasis | 8.76E-1±1.96E-1 | 7.226E-1±2.133E-1 | 7.99E-1±2.17E-1 | 0.009 | 0.902 |
| wavelet-LLL | glszm | SizeZoneNonUniformityNormalized | 8.3E-1±2.67E-1 | 5.814E-1±3.076E-1 | 7.06E-1±3.12E-1 | 0.004 | 0.859 |
| wavelet-LLL | glszm | ZoneEntropy | 3.76E-1±6.14E-1 | 1.098E+0±1.003E+0 | 7.37E-1±9.01E-1 | 0.003 | 0.826 |
| wavelet-LLL | glszm | ZoneVariance | 3.26E+2±8.52E+2 | 5.925E+3±1.427E+4 | 3.13E+3±1.04E+4 | 0.002 | 0.909 |
| wavelet-LLL | ngtdm | Complexity | 2.11E-1±4.E-1 | 1.347E+0±3.737E+0 | 7.79E-1±2.7E+0 | 0.017 | 0.98 |
| Pathological Complete Response (pCR); Spearman’s rank correlation coefficient (r_S_) | | | | | | | |
