# Supplementary material for: 18F-FDG PET/CT radiomic analysis and artificial intelligence to predict pathological complete response after neoadjuvant chemotherapy in breast cancer patients
Source: Radiol Med. 2025 Jan 28;130(4):543–54. doi: 10.1007/s11547-025-01958-4 (PMC12008070; doi:10.1007/s11547-025-01958-4)
Supplement: Supplementary file 3 — Supplementary file3 (DOCX 42 KB) [file 11547_2025_1958_MOESM3_ESM.docx]

**Table3S: Robust PET_T+N derived radiomic features.**

| Filter | Class | Radiomic feature | pCR1 | pCR0 | Tot | p | \|r_s_\| |
| --- | --- | --- | --- | --- | --- | --- | --- |
| original | Shape | Maximum2DDiameterRow | 2.49E+1±1.52E+1 | 9.94E+0±8.27E+0 | 2.77E+1±1.54E+1 | 0.025 | 0.839 |
| original | Shape | MeshVolume | 4.77E+3±8.7E+3 | 3.06E+1±1.52E+1 | 7.93E+3±1.37E+4 | 0.009 | 0.841 |
| original | Shape | SurfaceArea | 1.6E+3±2.22E+3 | 1.11E+4±1.69E+4 | 2.26E+3±3.09E+3 | 0.009 | 0.995 |
| original | Shape | SurfaceVolumeRatio | 5.2E-1±2.E-1 | 2.93E+3±3.68E+3 | 4.66E-1±1.98E-1 | 0.019 | 0.944 |
| original | Shape | VoxelVolume | 5.06E+3±8.88E+3 | 4.1E-1±1.8E-1 | 8.31E+3±1.4E+4 | 0.009 | 0.842 |
| original | FirstOrder | Maximum | 9.77E+0±6.32E+0 | 1.16E+4±1.73E+4 | 1.29E+1±1.1E+1 | 0.022 | 0.981 |
| original | FirstOrder | Mean | 4.91E+0±2.69E+0 | 1.6E+1±1.36E+1 | 6.06E+0±3.97E+0 | 0.03 | 0.923 |
| original | FirstOrder | RootMeanSquared | 5.31E+0±2.98E+0 | 7.21E+0±4.69E+0 | 6.66E+0±4.65E+0 | 0.025 | 0.874 |
| original | FirstOrder | GrayLevelNonUniformity | 1.02E+2±1.78E+2 | 8.01E+0±5.58E+0 | 1.58E+2±2.52E+2 | 0.012 | 0.961 |
| original | FirstOrder | LargeDependenceHighGrayLevelEmphasis | 2.01E+2±1.09E+2 | 2.14E+2±3.01E+2 | 2.34E+2±1.22E+2 | 0.019 | 0.875 |
| original | FirstOrder | GrayLevelNonUniformity | 3.9E+1±5.25E+1 | 2.68E+2±1.27E+2 | 5.65E+1±7.79E+1 | 0.008 | 0.938 |
| original | FirstOrder | RunEntropy | 1.5E+0±5.3E-1 | 7.39E+1±9.44E+1 | 1.69E+0±6.18E-1 | 0.007 | 0.868 |
| original | FirstOrder | RunLengthNonUniformity | 1.23E+1±9.79E+0 | 1.88E+0±6.4E-1 | 1.8E+1±2.47E+1 | 0.009 | 0.947 |
| original | FirstOrder | RunVariance | 1.08E+0±1.2E+0 | 2.37E+1±3.28E+1 | 1.39E+0±1.41E+0 | 0.018 | 0.891 |
| original | FirstOrder | LargeAreaEmphasis | 4.13E+4±1.69E+5 | 1.7E+0±1.54E+0 | 8.26E+4±2.53E+5 | 0.021 | 0.841 |
| original | FirstOrder | LargeAreaLowGrayLevelEmphasis | 4.13E+4±1.69E+5 | 1.24E+5±3.13E+5 | 8.2E+4±2.52E+5 | 0.021 | 0.862 |
| original | FirstOrder | ZonePercentage | 4.E-2±4.E-2 | 1.23E+5±3.1E+5 | 3.04E-2±3.58E-2 | 0.019 | 0.803 |
| wavelet-LLH | FirstOrder | Maximum | 1.67E+0±1.25E+0 | 2.E-2±3.E-2 | 2.38E+0±2.26E+0 | 0.026 | 0.864 |
| wavelet-LLH | glrlm | GrayLevelNonUniformity | 3.08E+1±5.17E+1 | 3.09E+0±2.79E+0 | 4.61E+1±7.13E+1 | 0.011 | 0.935 |
| wavelet-LLH | glrlm | RunEntropy | 2.05E+0±4.4E-1 | 6.13E+1±8.46E+1 | 2.2E+0±4.89E-1 | 0.004 | 0.802 |
| wavelet-LLH | glrlm | ShortRunLowGrayLevelEmphasis | 4.3E-1±8.E-2 | 2.35E+0±4.9E-1 | 4.08E-1±9.3E-2 | 0.024 | 0.807 |
| wavelet-LLH | glszm | LargeAreaHighGrayLevelEmphasis | 1.27E+4±5.87E+4 | 3.8E-1±1.E-1 | 3.01E+4±1.23E+5 | 0.008 | 0.836 |
| wavelet-LLH | ngtdm | Coarseness | 2.4E-1±2.1E-1 | 4.76E+4±1.63E+5 | 1.93E-1±1.95E-1 | 0.022 | 0.986 |
| wavelet-LLH | ngtdm | Strength | 2.3E-1±2.E-1 | 1.5E-1±1.7E-1 | 1.9E-1±1.91E-1 | 0.023 | 0.96 |
| wavelet-LHL | FirstOrder | Energy | 7.4E+2±1.57E+3 | 1.5E-1±1.7E-1 | 2.89E+3±1.01E+4 | 0.01 | 0.95 |
| wavelet-LHL | FirstOrder | Entropy | 9.3E-1±1.E-1 | 5.04E+3±1.39E+4 | 9.54E-1±9.19E-2 | 0.035 | 0.873 |
| wavelet-LHL | FirstOrder | TotalEnergy | 3.68E+4±7.79E+4 | 9.8E-1±8.E-2 | 1.44E+5±5.01E+5 | 0.01 | 0.95 |
| wavelet-LHL | FirstOrder | Uniformity | 5.5E-1±6.E-2 | 2.51E+5±6.92E+5 | 5.34E-1±5.01E-2 | 0.035 | 0.873 |
| wavelet-LHL | glcm | SumSquares | 1.5E-1±6.E-2 | 5.2E-1±3.E-2 | 1.7E-1±6.24E-2 | 0.021 | 0.871 |
| wavelet-LHL | gldm | DependenceNonUniformity | 8.91E+0±1.17E+1 | 1.9E-1±6.E-2 | 1.26E+1±1.77E+1 | 0.009 | 0.85 |
| wavelet-LHL | gldm | GrayLevelNonUniformity | 5.32E+1±9.11E+1 | 1.63E+1±2.17E+1 | 8.65E+1±1.43E+2 | 0.011 | 0.8 |
| wavelet-LHL | gldm | GrayLevelVariance | 2.3E-1±3.E-2 | 1.2E+2±1.76E+2 | 2.35E-1±3.17E-2 | 0.035 | 0.873 |
| wavelet-LHL | glrlm | GrayLevelNonUniformity | 3.17E+1±5.2E+1 | 2.4E-1±3.E-2 | 4.7E+1±7.12E+1 | 0.01 | 0.953 |
| wavelet-LHL | glrlm | GrayLevelNonUniformityNormalized | 5.3E-1±5.E-2 | 6.23E+1±8.43E+1 | 5.17E-1±3.99E-2 | 0.024 | 0.924 |
| wavelet-LHL | glrlm | GrayLevelVariance | 2.3E-1±2.E-2 | 5.E-1±2.E-2 | 2.44E-1±3.44E-2 | 0.024 | 0.924 |
| wavelet-LHL | glrlm | LongRunHighGrayLevelEmphasis | 7.79E+0±3.34E+0 | 2.5E-1±4.E-2 | 9.78E+0±7.84E+0 | 0.007 | 0.887 |
| wavelet-LHL | glrlm | RunLengthNonUniformityNormalized | 5.7E-1±1.5E-1 | 1.18E+1±1.03E+1 | 5.32E-1±1.67E-1 | 0.029 | 0.985 |
| wavelet-LHL | glrlm | RunPercentage | 7.E-1±1.1E-1 | 5.E-1±1.8E-1 | 6.71E-1±1.24E-1 | 0.035 | 0.808 |
| wavelet-LHL | glszm | LargeAreaLowGrayLevelEmphasis | 6.65E+3±3.07E+4 | 6.4E-1±1.3E-1 | 9.19E+3±3.13E+4 | 0.032 | 0.892 |
| wavelet-LHL | glszm | ZonePercentage | 8.E-2±8.E-2 | 1.17E+4±3.21E+4 | 6.39E-2±7.16E-2 | 0.046 | 0.938 |
| wavelet-LHL | ngtdm | Coarseness | 2.1E-1±2.2E-1 | 5.E-2±6.E-2 | 1.52E-1±1.81E-1 | 0.005 | 0.886 |
| wavelet-LHH | glcmD | DifferenceEntropy | 8.2E-1±2.1E-1 | 1.E-1±1.1E-1 | 8.56E-1±1.8E-1 | 0.038 | 0.812 |
| wavelet-LHH | glcmJ | JointEnergy | 3.8E-1±1.6E-1 | 8.9E-1±1.3E-1 | 3.48E-1±1.32E-1 | 0.01 | 0.996 |
| wavelet-LHH | glcmJ | JointEntropy | 1.6E+0±4.7E-1 | 3.2E-1±9.E-2 | 1.69E+0±4.04E-1 | 0.011 | 0.965 |
| wavelet-LHH | glcmS | SumEntropy | 1.22E+0±3.6E-1 | 1.79E+0±3.E-1 | 1.29E+0±3.16E-1 | 0.01 | 0.821 |
| wavelet-LHH | glcmS | SumSquares | 2.1E-1±5.E-2 | 1.36E+0±2.5E-1 | 2.21E-1±4.58E-2 | 0.003 | 0.912 |
| wavelet-LHH | gldmD | DependenceNonUniformity | 9.95E+0±1.35E+1 | 2.3E-1±3.E-2 | 1.49E+1±2.2E+1 | 0.008 | 0.895 |
| wavelet-LHH | gldmG | GrayLevelNonUniformity | 5.13E+1±8.91E+1 | 1.98E+1±2.73E+1 | 8.39E+1±1.41E+2 | 0.01 | 0.864 |
| wavelet-LHH | gldmL | LargeDependenceLowGrayLevelEmphasis | 4.E+1±2.53E+1 | 1.17E+2±1.74E+2 | 5.05E+1±2.94E+1 | 0.002 | 0.963 |
| wavelet-LHH | glrlm | GrayLevelNonUniformity | 3.42E+1±5.55E+1 | 6.09E+1±2.97E+1 | 5.3E+1±8.2E+1 | 0.008 | 0.97 |
| wavelet-LHH | glrlm | LongRunLowGrayLevelEmphasis | 1.46E+0±4.9E-1 | 7.18E+1±9.9E+1 | 1.67E+0±6.03E-1 | 0.003 | 0.922 |
| wavelet-LHH | glrlm | RunLengthNonUniformity | 3.54E+1±5.08E+1 | 1.88E+0±6.4E-1 | 5.14E+1±7.24E+1 | 0.008 | 0.906 |
| wavelet-LHH | glrlm | ShortRunEmphasis | 7.7E-1±8.E-2 | 6.74E+1±8.66E+1 | 7.43E-1±8.2E-2 | 0.014 | 0.919 |
| wavelet-LHH | glrlm | ShortRunLowGrayLevelEmphasis | 4.8E-1±6.E-2 | 7.2E-1±8.E-2 | 4.68E-1±5.98E-2 | 0.005 | 0.839 |
| wavelet-LHH | glszm | LargeAreaEmphasis | 7.55E+3±2.81E+4 | 4.5E-1±6.E-2 | 1.77E+4±5.93E+4 | 0.011 | 0.903 |
| wavelet-LHH | glszm | LargeAreaHighGrayLevelEmphasis | 1.86E+4±7.E+4 | 2.79E+4±7.83E+4 | 4.27E+4±1.42E+5 | 0.011 | 0.979 |
| wavelet-LHH | glszm | ZonePercentage | 9.E-2±9.E-2 | 6.69E+4±1.87E+5 | 6.61E-2±7.92E-2 | 0.008 | 0.882 |
| wavelet-LHH | ngtdm | Coarseness | 1.6E-1±1.7E-1 | 5.E-2±6.E-2 | 1.22E-1±1.44E-1 | 0.007 | 0.886 |
| wavelet-LHH | ngtdm | Strength | 1.6E-1±1.6E-1 | 9.E-2±1.1E-1 | 1.21E-1±1.43E-1 | 0.007 | 0.957 |
| wavelet-HLL | FirstOrder | Energy | -3.7E+0±3.08E+0 | 9.E-2±1.1E-1 | -4.7E+0±4.12E+0 | 0.025 | 0.858 |
| wavelet-HLL | FirstOrder | Entropy | 9.2E-1±1.7E-1 | -5.71E+0±4.78E+0 | 9.55E-1±1.39E-1 | 0.037 | 0.865 |
| wavelet-HLL | FirstOrder | RootMeanSquared | 2.49E+0±1.79E+0 | 9.9E-1±9.E-2 | 3.05E+0±2.45E+0 | 0.049 | 0.956 |
| wavelet-HLL | FirstOrder | Variance | 8.56E+0±1.35E+1 | 3.61E+0±2.88E+0 | 1.42E+1±2.76E+1 | 0.037 | 0.979 |
| wavelet-HLL | glcmS | SumEntropy | 1.04E+0±4.6E-1 | 1.97E+1±3.6E+1 | 1.14E+0±4.38E-1 | 0.006 | 0.816 |
| wavelet-HLL | glcmS | SumSquares | 1.6E-1±7.E-2 | 1.25E+0±4.E-1 | 1.79E-1±7.32E-2 | 0.002 | 0.803 |
| wavelet-HLL | gldmD | DependenceNonUniformity | 8.62E+0±1.04E+1 | 2.E-1±7.E-2 | 1.26E+1±1.75E+1 | 0.004 | 0.932 |
| wavelet-HLL | gldmG | GrayLevelVariance | 2.3E-1±4.E-2 | 1.67E+1±2.18E+1 | 2.38E-1±4.53E-2 | 0.037 | 0.865 |
| wavelet-HLL | gldmL | LargeDependenceHighGrayLevelEmphasis | 1.63E+2±9.82E+1 | 2.5E-1±4.E-2 | 2.59E+2±2.91E+2 | 0.002 | 0.963 |
| wavelet-HLL | glrlm | GrayLevelNonUniformity | 3.17E+1±4.96E+1 | 3.55E+2±3.78E+2 | 4.78E+1±7.2E+1 | 0.011 | 0.952 |
| wavelet-HLL | glrlm | RunEntropy | 1.88E+0±5.1E-1 | 6.39E+1±8.68E+1 | 2.05E+0±5.48E-1 | 0.009 | 0.84 |
| wavelet-HLL | glszm | LargeAreaHighGrayLevelEmphasis | 1.17E+4±4.52E+4 | 2.22E+0±5.4E-1 | 3.58E+4±1.33E+5 | 0.008 | 0.878 |
| wavelet-HLL | glszm | ZonePercentage | 8.E-2±8.E-2 | 6.E+4±1.8E+5 | 6.28E-2±6.9E-2 | 0.021 | 0.919 |
| wavelet-HLH | glcmI | Imc1 | -1.3E-1±1.1E-1 | 5.E-2±6.E-2 | -1.09E-1±9.81E-2 | 0.007 | 0.854 |
| wavelet-HLH | glcmI | Imc2 | 3.E-1±9.E-2 | -8.E-2±7.E-2 | 2.78E-1±9.34E-2 | 0.019 | 0.846 |
| wavelet-HLH | glcm | MCC | 2.7E-1±9.E-2 | 2.6E-1±9.E-2 | 2.48E-1±9.23E-2 | 0.015 | 0.989 |
| wavelet-HLH | glcm | SumEntropy | 1.17E+0±4.2E-1 | 2.2E-1±8.E-2 | 1.26E+0±3.71E-1 | 0.004 | 0.894 |
| wavelet-HLH | gldm | DependenceEntropy | 3.62E+0±1.02E+0 | 1.34E+0±3.E-1 | 3.86E+0±9.93E-1 | 0.007 | 0.865 |
| wavelet-HLH | gldm | GrayLevelNonUniformity | 5.14E+1±8.91E+1 | 4.09E+0±9.2E-1 | 8.41E+1±1.41E+2 | 0.008 | 0.936 |
| wavelet-HLH | gldm | HighGrayLevelEmphasis | 2.5E+0±2.4E-1 | 1.17E+2±1.74E+2 | 2.44E+0±2.46E-1 | 0.046 | 0.966 |
| wavelet-HLH | gldm | LargeDependenceEmphasis | 6.73E+1±3.51E+1 | 2.39E+0±2.4E-1 | 7.95E+1±4.1E+1 | 0.014 | 0.958 |
| wavelet-HLH | gldm | LowGrayLevelEmphasis | 6.2E-1±6.E-2 | 9.17E+1±4.33E+1 | 6.39E-1±6.14E-2 | 0.046 | 0.966 |
| wavelet-HLH | gldm | SmallDependenceEmphasis | 4.E-2±3.E-2 | 6.5E-1±6.E-2 | 3.74E-2±3.2E-2 | 0.02 | 0.89 |
| wavelet-HLH | gldm | SmallDependenceHighGrayLevelEmphasis | 1.E-1±1.E-1 | 3.E-2±3.E-2 | 1.01E-1±1.07E-1 | 0.021 | 0.897 |
| wavelet-HLH | gldm | SmallDependenceLowGrayLevelEmphasis | 2.E-2±2.E-2 | 1.E-1±1.2E-1 | 2.15E-2±1.5E-2 | 0.026 | 0.845 |
| wavelet-HLH | glrlm | GrayLevelNonUniformity | 3.45E+1±5.58E+1 | 2.E-2±1.E-2 | 5.36E+1±8.31E+1 | 0.008 | 0.915 |
| wavelet-HLH | glrlm | LongRunEmphasis | 2.4E+0±6.4E-1 | 7.27E+1±1.01E+2 | 2.65E+0±8.4E-1 | 0.02 | 0.929 |
| wavelet-HLH | glrlm | LongRunLowGrayLevelEmphasis | 1.49E+0±4.4E-1 | 2.89E+0±9.4E-1 | 1.69E+0±5.61E-1 | 0.002 | 0.824 |
| wavelet-HLH | glrlm | RunEntropy | 1.89E+0±3.6E-1 | 1.89E+0±6.E-1 | 2.E+0±3.91E-1 | 0.008 | 0.962 |
| wavelet-HLH | glrlm | RunLengthNonUniformity | 3.61E+1±5.13E+1 | 2.11E+0±4.E-1 | 5.26E+1±7.48E+1 | 0.011 | 0.95 |
| wavelet-HLH | glrlm | RunLengthNonUniformityNormalized | 6.1E-1±1.1E-1 | 6.91E+1±9.02E+1 | 5.75E-1±1.18E-1 | 0.011 | 0.873 |
| wavelet-HLH | glrlm | RunPercentage | 7.5E-1±8.E-2 | 5.4E-1±1.1E-1 | 7.26E-1±8.76E-2 | 0.012 | 0.906 |
| wavelet-HLH | glrlm | RunVariance | 3.7E-1±2.1E-1 | 7.E-1±9.E-2 | 4.57E-1±2.85E-1 | 0.009 | 0.871 |
| wavelet-HLH | glrlm | ShortRunEmphasis | 7.6E-1±7.E-2 | 5.4E-1±3.2E-1 | 7.44E-1±7.79E-2 | 0.023 | 0.923 |
| wavelet-HLH | ngtdm | Strength | 1.7E-1±1.8E-1 | 7.2E-1±8.E-2 | 1.3E-1±1.66E-1 | 0.01 | 0.802 |
| wavelet-HHL | FirstOrder | Minimum | -1.61E+0±1.42E+0 | 9.E-2±1.4E-1 | -2.07E+0±2.08E+0 | 0.047 | 0.948 |
| wavelet-HHL | glcm | ClusterTendency | 3.9E-1±1.4E-1 | -2.52E+0±2.52E+0 | 4.23E-1±1.31E-1 | 0.01 | 0.976 |
| wavelet-HHL | glcm | SumSquares | 1.9E-1±7.E-2 | 4.5E-1±1.2E-1 | 2.06E-1±5.84E-2 | 0.049 | 0.827 |
| wavelet-HHL | gldm | DependenceEntropy | 3.54E+0±1.07E+0 | 2.2E-1±5.E-2 | 3.81E+0±1.02E+0 | 0.005 | 0.929 |
| wavelet-HHL | gldm | LargeDependenceEmphasis | 6.86E+1±3.42E+1 | 4.07E+0±9.1E-1 | 7.93E+1±3.99E+1 | 0.016 | 0.8 |
| wavelet-HHL | gldm | SmallDependenceEmphasis | 4.E-2±3.E-2 | 9.01E+1±4.27E+1 | 3.76E-2±2.95E-2 | 0.013 | 0.853 |
| wavelet-HHL | gldm | SmallDependenceHighGrayLevelEmphasis | 1.1E-1±9.E-2 | 3.E-2±3.E-2 | 9.81E-2±9.37E-2 | 0.025 | 0.946 |
| wavelet-HHL | glrlm | GrayLevelNonUniformity | 3.48E+1±5.64E+1 | 9.E-2±1.E-1 | 5.4E+1±8.35E+1 | 0.007 | 0.873 |
| wavelet-HHL | glrlm | LongRunEmphasis | 2.7E+0±7.6E-1 | 7.31E+1±1.01E+2 | 2.99E+0±9.77E-1 | 0.022 | 0.807 |
| wavelet-HHL | glrlm | LongRunLowGrayLevelEmphasis | 1.59E+0±5.8E-1 | 3.27E+0±1.09E+0 | 1.83E+0±7.32E-1 | 0.005 | 0.851 |
| wavelet-HHL | glrlm | RunEntropy | 1.85E+0±3.8E-1 | 2.08E+0±7.9E-1 | 1.96E+0±4.14E-1 | 0.018 | 0.958 |
| wavelet-HHL | glrlm | RunLengthNonUniformity | 3.62E+1±5.25E+1 | 2.07E+0±4.2E-1 | 5.32E+1±7.49E+1 | 0.006 | 0.924 |
| wavelet-HHL | glrlm | RunPercentage | 7.5E-1±8.E-2 | 7.02E+1±8.96E+1 | 7.26E-1±8.51E-2 | 0.015 | 0.825 |
| wavelet-HHL | glrlm | RunVariance | 4.7E-1±2.6E-1 | 7.E-1±9.E-2 | 5.73E-1±3.54E-1 | 0.032 | 0.944 |
| wavelet-HHL | glszm | LargeAreaLowGrayLevelEmphasis | 3.64E+3±1.19E+4 | 6.7E-1±4.1E-1 | 1.36E+4±5.51E+4 | 0.007 | 0.838 |
| wavelet-HHL | glszm | SmallAreaHighGrayLevelEmphasis | 3.E-1±5.E-1 | 2.36E+4±7.62E+4 | 2.66E-1±5.47E-1 | 0.012 | 0.814 |
| wavelet-HHL | glszm | ZonePercentage | 9.E-2±8.E-2 | 2.3E-1±6.E-1 | 6.49E-2±7.39E-2 | 0.005 | 0.953 |
| wavelet-HHL | ngtdm | Strength | 1.7E-1±1.8E-1 | 4.E-2±6.E-2 | 1.33E-1±1.81E-1 | 0.006 | 0.818 |
| wavelet-HHH | glcm | SumSquares | 2.1E-1±6.E-2 | 1.E-1±1.7E-1 | 2.24E-1±4.9E-2 | 0.008 | 0.98 |
| wavelet-HHH | gldm | DependenceNonUniformityNormalized | 1.9E-1±1.2E-1 | 2.4E-1±3.E-2 | 1.62E-1±1.13E-1 | 0.01 | 0.83 |
| wavelet-HHH | gldm | GrayLevelNonUniformity | 5.11E+1±8.95E+1 | 1.4E-1±1.E-1 | 8.37E+1±1.41E+2 | 0.008 | 0.83 |
| wavelet-HHH | gldm | SmallDependenceHighGrayLevelEmphasis | 1.E-1±8.E-2 | 1.16E+2±1.73E+2 | 9.32E-2±8.41E-2 | 0.029 | 0.938 |
| wavelet-HHH | glrlm | GrayLevelNonUniformity | 3.54E+1±5.78E+1 | 8.E-2±9.E-2 | 5.56E+1±8.71E+1 | 0.008 | 0.95 |
| wavelet-HHH | glrlm | LongRunHighGrayLevelEmphasis | 5.36E+0±1.48E+0 | 7.58E+1±1.06E+2 | 5.88E+0±1.73E+0 | 0.011 | 0.874 |
| wavelet-HHH | glrlm | RunLengthNonUniformity | 3.8E+1±5.51E+1 | 6.4E+0±1.82E+0 | 5.66E+1±8.15E+1 | 0.008 | 0.934 |
| wavelet-HHH | glrlm | RunLengthNonUniformityNormalized | 6.2E-1±1.E-1 | 7.53E+1±9.85E+1 | 5.92E-1±1.03E-1 | 0.011 | 0.836 |
| wavelet-HHH | glrlm | RunPercentage | 7.7E-1±7.E-2 | 5.6E-1±1.E-1 | 7.46E-1±8.01E-2 | 0.008 | 0.822 |
| wavelet-HHH | glrlm | ShortRunHighGrayLevelEmphasis | 1.97E+0±1.7E-1 | 7.2E-1±8.E-2 | 1.93E+0±2.01E-1 | 0.011 | 0.8 |
| wavelet-HHH | ngtdm | Coarseness | 1.5E-1±1.6E-1 | 1.89E+0±2.2E-1 | 1.17E-1±1.47E-1 | 0.008 | 0.997 |
| wavelet-HHH | ngtdm | Strength | 1.5E-1±1.6E-1 | 8.E-2±1.2E-1 | 1.17E-1±1.45E-1 | 0.008 | 0.965 |
| wavelet-LLL | FirstOrder |  | 1.81E+1±1.1E+1 | 8.E-2±1.2E-1 | 2.43E+1±2.E+1 | 0.011 | 0.977 |
| wavelet-LLL | FirstOrder | InterquartileRange | 5.61E+0±4.52E+0 | 3.05E+1±2.46E+1 | 8.55E+0±9.75E+0 | 0.02 | 0.99 |
| wavelet-LLL | FirstOrder | Maximum | 2.23E+1±1.47E+1 | 1.15E+1±1.24E+1 | 3.02E+1±2.6E+1 | 0.016 | 0.96 |
| wavelet-LLL | FirstOrder | MeanAbsoluteDeviation | 3.31E+0±2.56E+0 | 3.82E+1±3.21E+1 | 4.88E+0±5.29E+0 | 0.031 | 0.876 |
| wavelet-LLL | FirstOrder | Mean | 1.27E+1±7.2E+0 | 6.44E+0±6.73E+0 | 1.6E+1±1.11E+1 | 0.015 | 0.893 |
| wavelet-LLL | FirstOrder | Median | 1.22E+1±6.93E+0 | 1.94E+1±1.32E+1 | 1.52E+1±1.01E+1 | 0.02 | 0.905 |
| wavelet-LLL | FirstOrder | Range | 1.65E+1±1.36E+1 | 1.81E+1±1.19E+1 | 2.41E+1±2.53E+1 | 0.024 | 0.801 |
| wavelet-LLL | FirstOrder | RobustMeanAbsoluteDeviation | 2.38E+0±1.89E+0 | 3.18E+1±3.15E+1 | 3.59E+0±4.04E+0 | 0.025 | 0.981 |
| wavelet-LLL | FirstOrder | RootMeanSquared | 1.33E+1±7.74E+0 | 4.79E+0±5.15E+0 | 1.72E+1±1.26E+1 | 0.012 | 0.89 |
| wavelet-LLL | FirstOrder | Variance | 2.55E+1±3.67E+1 | 2.11E+1±1.51E+1 | 7.27E+1±1.96E+2 | 0.041 | 0.83 |
| wavelet-LLL | gldm | HighGrayLevelEmphasis | 1.27E+0±5.7E-1 | 1.2E+2±2.68E+2 | 1.73E+0±1.65E+0 | 0.001 | 0.804 |
| wavelet-LLL | gldm | SmallDependenceHighGrayLevelEmphasis | 2.E-2±2.E-2 | 2.2E+0±2.17E+0 | 3.96E-2±9.08E-2 | 0.026 | 0.852 |
| wavelet-LLL | glrlm | GrayLevelNonUniformity | 3.81E+1±5.25E+1 | 6.E-2±1.2E-1 | 5.4E+1±7.54E+1 | 0.019 | 0.996 |
| wavelet-LLL | glrlm | RunLengthNonUniformity | 1.65E+1±1.43E+1 | 6.99E+1±9.08E+1 | 3.31E+1±6.09E+1 | 0.001 | 0.833 |
| wavelet-LLL | glrlm | ShortRunHighGrayLevelEmphasis | 7.2E-1±3.9E-1 | 4.98E+1±8.21E+1 | 1.03E+0±1.29E+0 | 0.008 | 0.901 |
| wavelet-LLL | glszm | LargeAreaHighGrayLevelEmphasis | 3.87E+4±1.69E+5 | 1.33E+0±1.74E+0 | 5.37E+4±1.89E+5 | 0.032 | 0.962 |
| wavelet-LLL | ngtdm | Complexity | 1.6E-1±3.5E-1 | 6.87E+4±2.08E+5 | 6.39E-1±2.35E+0 | 0.003 | 0.801 |
| Pathological Complete Response (pCR); Spearman’s rank correlation coefficient (r_S_). | | | | | | | |
