# Supplementary material for: 18F-FDG PET/CT radiomic analysis and artificial intelligence to predict pathological complete response after neoadjuvant chemotherapy in breast cancer patients
Source: Radiol Med. 2025 Jan 28;130(4):543–54. doi: 10.1007/s11547-025-01958-4 (PMC12008070; doi:10.1007/s11547-025-01958-4)

**Figure S1.** Comparison of the performances scores of the learners for each of the ML model trained. a Clinical Model, b CT Model, c PET Model_T, d PET Model_T+N.


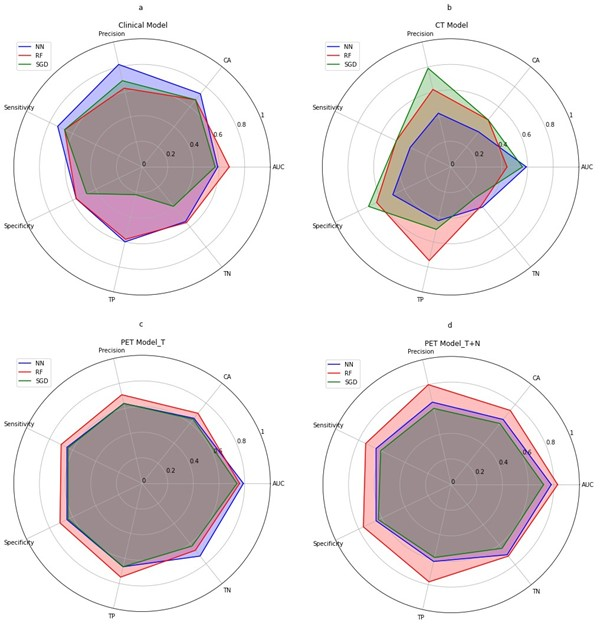

Supplement: Supplementary file 4 — Supplementary file4 (DOCX 329 KB) [file 11547_2025_1958_MOESM4_ESM.docx]
